# Loss of Autonoetic Awareness of Recent Autobiographical Episodes and Accelerated Long-Term Forgetting in a Patient with Previously Unrecognized Glutamic Acid Decarboxylase Antibody Related Limbic Encephalitis

**DOI:** 10.3389/fneur.2015.00130

**Published:** 2015-06-09

**Authors:** Juri-Alexander Witt, Viola Lara Vogt, Guido Widman, Karl-Josef Langen, Christian Erich Elger, Christoph Helmstaedter

**Affiliations:** ^1^Department of Epileptology, University of Bonn, Bonn, Germany; ^2^Institute of Neuroscience and Medicine, Forschungszentrum Jülich, Jülich, Germany

**Keywords:** autoimmune encephalitis, autoantibody, accelerated forgetting, memory, cognition, neuropsychology, epilepsy, amygdala

## Abstract

We describe a 35-year-old male patient presenting with depressed mood and emotional instability, who complained about severe anterograde and retrograde memory deficits characterized by accelerated long-term forgetting and loss of autonoetic awareness regarding autobiographical memories of the last 3 years. Months before he had experienced two breakdowns of unknown etiology giving rise to the differential diagnosis of epileptic seizures after various practitioners and clinics had suggested different etiologies such as a psychosomatic condition, burnout, depression, or dissociative amnesia. Neuropsychological assessment indicated selectively impaired figural memory performance. Extended diagnostics confirmed accelerated forgetting of previously learned and retrievable verbal material. Structural imaging showed bilateral swelling and signal alterations of temporomesial structures (left >right). Video-EEG monitoring revealed a left temporal epileptic focus and subclincal seizure, but no overt seizures. Antibody tests in serum and liquor were positive for glutamic acid decarboxylase antibodies. These findings led to the diagnosis of glutamic acid decarboxylase antibody related limbic encephalitis. Monthly steroid pulses over 6 months led to recovery of subjective memory and to intermediate improvement but subsequent worsening of objective memory performance. During the course of treatment, the patient reported *de novo* paroxysmal non-responsive states. Thus, antiepileptic treatment was started and the patient finally became seizure free. At the last visit, vocational reintegration was successfully in progress. In conclusion, amygdala swelling, retrograde biographic memory impairment, accelerated long-term forgetting, and emotional instability may serve as indicators of limbic encephalitis, even in the absence of overt epileptic seizures. The monitoring of such patients calls for a standardized and concerted multilevel diagnostic approach with repeated assessments.

## Introduction

Limbic encephalitis defines a clinical syndrome caused by autoimmune-mediated structural–morphological and metabolic changes primarily affecting mesiotemporal structures (1, 2). Thus, clinical symptoms can comprise episodic memory impairment, affective disturbances, and/or temporal lobe seizures. Paraneoplastic and non-paraneoplastic subforms of limbic encephalitis are differentiated, and meanwhile several autoantibodies underlying this autoimmune process have been identified (3–5). Autoantibodies can be directed against neuronal cell surface antigens or against intracellular neuronal antigens. Herewith, we report a patient with previously unrecognized glutamic acid decarboxylase (GAD) antibody related limbic encephalitis primarily characterized by severe neuropsychological impairments. Antibodies to the 65 kDa isoform of the intracellular enzyme GAD define a form of non-paraneoplastic limbic encephalitis (6).

## Case Report

The 35-year-old male patient consulted our outpatient department in February 2012, after having experienced a second episode of a breakdown with confusion and severe but unspecified headache. A first episode happened in September 2011, the second in January 2012. In the morning after the first episode, he realized a retrograde episodic memory loss spanning the preceding 3 years. For instance, he only had shadowy memories of a recent travel abroad without any emotional attachment or autonoetic awareness. The same was true for usually highly emotional incidents such as a wedding and a bereavement. In addition, as a teacher, he was no longer aware of the content of his last given lessons, but he was able to recall his pupils’ names and faces. Finally, anterograde memory problems were complained resembling the phenomenon of accelerated long-term forgetting, i.e., new information can be successfully learned and recalled, but after few days respective memory recall is no longer possible.

Since the first episode and before coming to our department, the patient went to various practitioners and clinics suggesting different etiologies such as a psychosomatic condition, a burnout, a depression, or dissociative amnesia. He presented with depressed mood, significant emotional instability, and bewilderment. He felt that he was not capable to work in this condition. The patient suffered from diabetes mellitus type 1 and this had also been considered as a possible source of his problems. It is important to note that there were no overt epileptic seizures at that time, but the patient reported brief aura-like states with an ascending feeling and an autoscopic experience (about twice per month), which he had interpreted as possibly resulting from hypoglycemia in the context of his diabetes.

**Figure 1:**
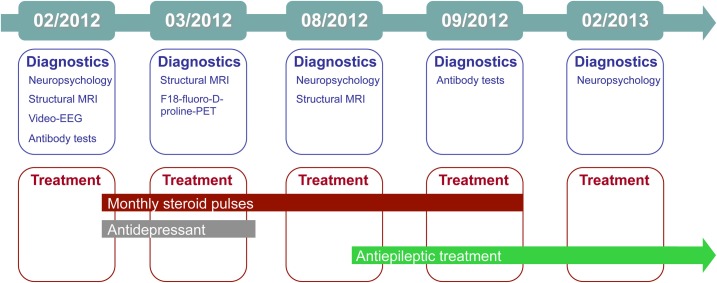
**Graphical overview of the chronology of diagnostics and treatments**.

The treating physician and the neuropsychologist who talked to the patient at our outpatient clinic suspected limbic encephalitis, and in the same month the patient underwent (differential) diagnostics as an in-patient. Figure 1 gives an graphical overview of the chronology of diagnostics and treatments. Complementary Table 1 summarizes the major findings.

**Table 1:** **Chronology and major findings in the course of the presented patient with glutamic acid decarboxylase antibody related limbic encephalitis**.

	September 2011	January 2012	February 2012	April and May 2012	August 2012	September 2012	September 2013
Comment	Breakdown	Breakdown	First outpatient contact with subsequent inpatient evaluation	–	–	–	Final contact; stepwise vocational reintegration in progress
Structural-morphological findings	–	–	Bilateral swelling + signal alterations of temporomesial structures (left >right)	–	Hippocampal volumes decreased within the normal range, enlargement of left amygdala remained	–	–
Objective memory	–	–	Verbal: average (30 min retention), but ALF (1 week retention); figural: impaired	–	Improvement Verbal: good Figural: borderline	–	Verbal: borderline Figural: impaired
Subjective memory	Anterograde and retrograde deficits	Anterograde and retrograde deficits	Anterograde and retrograde deficits	Clear anterograde memory improvement	Nearly premorbid level regarding anterograde memory	Memory decline	Memory improvement; partial recovery of biographical memory
Mood	?	?	Depressed mood	–	Depressed mood		Improved mood
			Emotional instability	
Seizures and epileptiform discharges	?	?	Left temporal focus in video-EEG	–	*De novo* non-responsive states	Two episodes of a strange feeling with subsequent memory loss	Unspecific feelings (aura?), no overt seizures
Titers	–	–	liquor (IFT): GAD 65: 1:3.2 serum (ELISA):GAD 65: >2.000 IE/ml			Liquor (IFT):GAD 65: 1:3.2 serum (ELISA):GAD 65: > 2.000 IE/ml	–
Treatment	–	–	Monthly steroid-pulses; antidepressant treatment	Monthly steroid-pulses	Monthly steroid-pulses; antiepileptic treatment	Last steroid-pulse; antiepileptic treatment	Antiepileptic treatment

*ALF, accelerated long-term forgetting; GAD, glutamic acid decarboxylase*.

**Figure 2:**
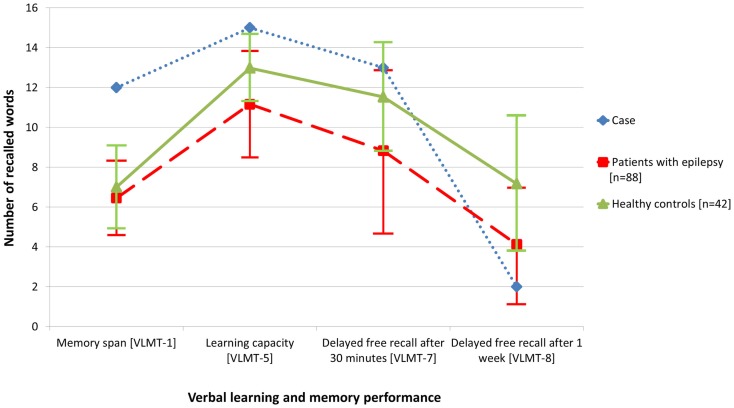
**Compared with data from healthy controls (*n* = 42), objective memory assessment in the presented patient with glutamic acid decarboxylase antibody related limbic encephalitis (February 2012) revealed normal to above average performance with regard to verbal learning and free recall after 30 min, but accelerated long-term forgetting after 1 week**. For comparison, the figure also illustrates the memory performance of a random sample of patients with epilepsy (*n* = 88).

The initial standardized neuropsychological assessment (February 2012) in the ambidextrous patient pointed to an impairment of figural learning (DCS-R, (7), whereas verbal learning and memory including delayed free recall after 30 min. (VLMT) (8) was normal, the learning performance even above average. Average results were achieved for attention and executive functions (EpiTrack^®^) (9). Based on this neuropsychological profile, a right temporal lobe dysfunction was deduced assuming typical left hemispheric language dominance (7, 10, 11). However, because of ambidexterity and left-hemispheric accentuation of the temporomesial structural abnormalities, language dominance was assessed by functional MRI (12) and functional transcranial Doppler sonography (13) concordantly indicating typical left hemispheric language dominance. In order to clarify the mismatch of subjective memory complaints and testing of verbal learning and memory and to address the subjective complaints of accelerated long-term forgetting, verbal memory material presented during the neuropsychological evaluation was requested again (free recall and recognition) 1 week later [cf. Ref. (14)]. Memory performance after this extended retention interval was severely impaired (Figure 2). In this way, the respective subjective complaints could be objectified.

The Beck depression inventory I indicated mildly depressed mood (13 points; cutoff >10).

**Figure 3:**
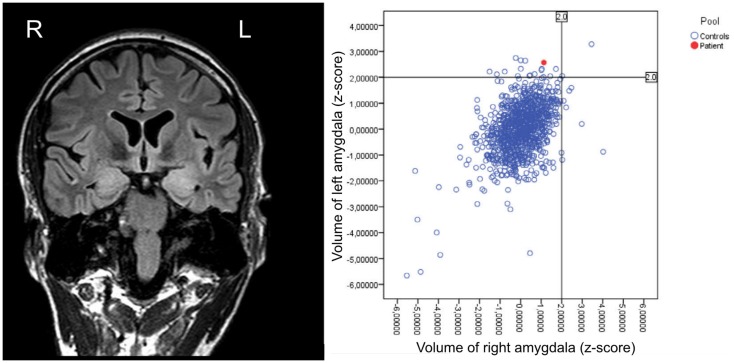
**Structural MRI (performed in February 2012) showing bilateral swelling with signal alterations of the temporomesial structures more prominent on the left hemisphere, and respective volumetric results revealing enlargement of the left amygdala as compared to healthy controls**.

Structural imaging showed a bilateral swelling with signal alterations of the temporomesial structures more prominent within the left hemisphere (Figure 3). MRI volumetry confirmed a significantly increased volume of the left amygdala as compared to normative data (Figure 3).

**Figure 4:**
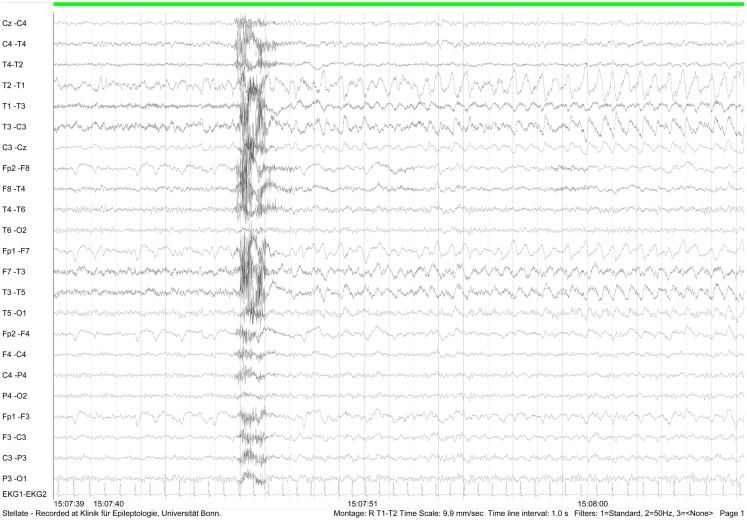
**EEG recording (February 2012) showing subclinical left frontotemporal seizure following hyperventilation**.

During video-EEG over five consecutive days, a specific left temporal focus and a subclinical seizure following hyperventilation were recorded (Figure 4).

Laboratory tests revealed oligoclonal bands and no evidence of blood–brain barrier disruption. Serum and liquor antibody tests were negative for amphiphsyin, CV2, Ma/Ta, Ri, Yo, and Hu, MAG, Tr, Myelin, Aquaporin, Glycin, NMDA, AMPA, GABAb, VGKC (incl. LGI1 and CASPR2), but positive for GAD 65 (liquor: 1:3,2 [IFT]) (serum: >2.000 IE/ml [ELISA]) and 67 (serum: 1:1.000 [IFT]) and varicella-zoster virus (VZV-IgG: serum: 3.600 mIE/ml [ELISA]). HHV6-IFT IgG und IgM were positive in serum [IFT].

Thorough tumor diagnostics were negative.

The overall findings led to the diagnosis of GAD antibody related limbic encephalitis and therefore immunotherapy was initiated comprising monthly steroid-pulse-therapy from February to September 2012 (1000 mg methylprednisolone i.v. on five consecutive days per cycle).

**Figure 5:**
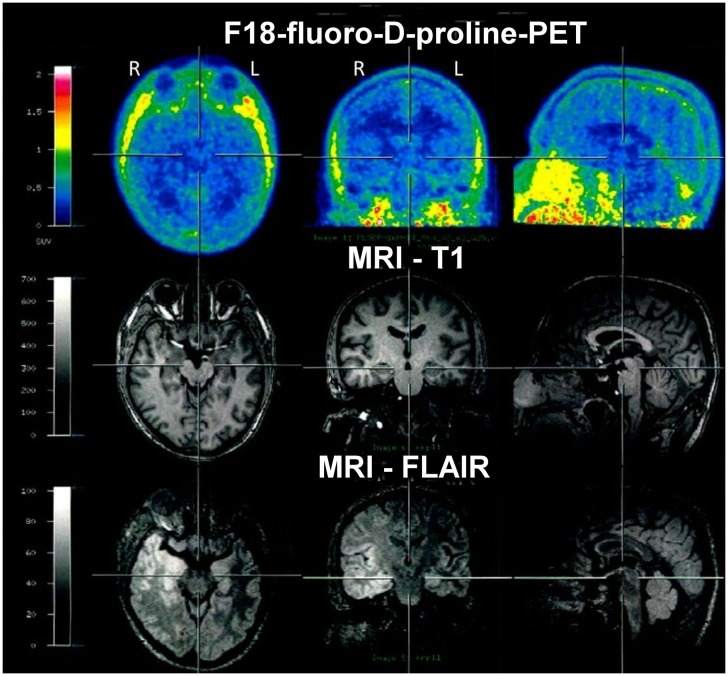
**F18-fluoro-d-proline-positron emission tomography (PET) in March 2012 showed no abnormal tracer accumulation**. Structural MRI again showed bitemporal hyperintensities, however, at that time clearly more marked within the right hemisphere.

In March 2012, F18-fluoro-d-proline-positron emission tomography (PET) was performed in search of biomarkers of an active inflammatory process. Studies had revealed that Cis-4-[F18]fluoro-d-proline uptake is associated with secondary neurodegeneration in the presence of brain tumors and after cortical infarction (15, 16). However, in the present case, an abnormal tracer accumulation was not observed (Figure 5). In contrast to structural imaging in February, the structural MRI at the time of the PET (for the purpose of image fusing) showed bitemporal hyperintensities now clearly more marked within the right hemisphere (Figure 5).

**Figure 6:**
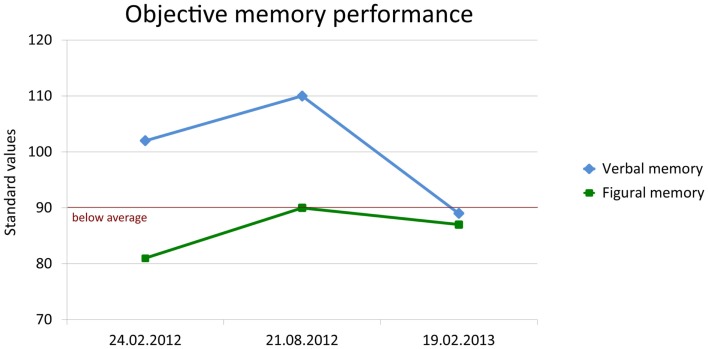
**Change of objective verbal (delayed free recall after 30 min) and figural (learning performance across five trials) memory performance**. Memory was standardized (standard values; M = 100, SD = 10) according to a normative sample of 488 healthy volunteers who underwent both tests for co-normalization.

In August, the patient reported *de novo* seizure-like non-responsive states. Therefore, antiepileptic treatment with lamotrigine (target dose 200 mg/d) was initiated. Repeated structural MRI with subsequent volumetry still showed an enlargement of the left amygdala, while the hippocampal volumes decreased but remained within the normal range of healthy subjects. The neuropsychological follow-up demonstrated significant improvement in figural memory performance when taking practice corrected reliable change indices as the reference for judging intraindividual change (*p* < 0.1) (Figure 6: first follow-up 21 August, 2012).

Verbal learning and memory improved as well, but taking into consideration the already good performance at baseline a ceiling effect was evident. Attention and executive functions were stable and remained unimpaired. The patient reported that, after the third cycle of steroid-pulse therapy in April, his memory improved to a nearly premorbid level. Episodes during the preceding month could be remembered without difficulty and there was no more self-perceived accelerated long-term forgetting. However, the Beck depression inventory still indicated depressed mood.

In September, before the sixth and last cycle of steroid-pulse therapy, the patient reported two episodes of a strange feeling with subsequent memory loss. Subjectively, memory performance had become worse. After the last cycle of immunotherapy, the patient was released in a good overall condition. No need for further immunotherapy was concluded.

One year after the first contact, in February 2013, the patient still reported infrequent episodes of unspecific feelings without loss of consciousness. The objective memory assessment now showed a significant decrease in verbal memory capacity and a borderline performance in figural memory (Figure 6: second follow-up 19 February, 2013). In contrast, subjective memory performance improved including a partial recovery of autobiographical memory and of mood as indicated by the depression inventory (BDI I: 6 points; cutoff: >10). Stepwise vocational reintegration (status at that time 75%) was successfully in progress.

## Discussion

The presented case report of a patient with previously unrecognized glutamic acid decarboxylase antibody related limbic encephalitis is noteworthy in several ways: first of all, the neuropsychological and psychiatric disturbances putatively preceded the first overt epileptic seizure, assuming that the breakdowns of unknown etiology had probably not been of epileptic origin. For some months, the patient underwent some sort of odyssey visiting various practitioners and clinics entailing different diagnoses ranging from burnout to dissociative amnesia. Overt epileptic seizures first manifested in the course of the immunotherapy. The neuropsychological memory profile was exceptional both in regard to anterograde and retrograde memory functions: retrograde memory deficits were characterized by loss of emotional attachment and autonoetic awareness of autobiographical memories spanning the preceding 3 years. Even a wedding and bereavement could not be ecphorized. There was a lack of an emotional attachment, the usually highly emotional events could only be remembered in a shadowy way and primarily on a semantic level. The affection of the left amygdala as revealed by structural imaging may have contributed to or even caused this reversible autobiographical memory impairment, since the amygdala is part of the basolateral limbic circuit which is relevant for emotional valence of memories (17). Unfortunately, only two structural MRIs have been conducted during the course of the disease both showing a swelling of the left amygdala which was confirmed by volumetry. However, at the final visit when partial recovery of the autobiographical memories was reported, no structural imaging was performed again to examine whether the swelling of the amygdala was regressive or even vanished. A recent longitudinal study by Wagner et al. described distinct volumetric and clinical courses in limbic encephalitis (18). Patients with GAD antibodies initially exhibited greater amygdala volumes with subsequent normalization in the course of treatment. Few studies reported retrograde memory deficits in limbic encephalitis with mostly bilateral affection of the mesiotemporal structures (19–23), but – to our knowledge – none of the investigated patients exhibited this specific loss of emotional attachment and autonoetic awareness. Moreover, none of the patients suffered from glutamic acid decarboxylase antibody related limbic encephalitis.

Besides his psychiatric problems, a major reason for the patient’s inability to keep working as a teacher was his severe anterograde memory impairment. Newly encoded episodes, e.g., the content of his last given lessons, could be remembered up to 3 days, but were then lost or no longer accessible. In a case report, De Renzi and Lucchelli (24) describe a patient with hypoxic brain injury who suffered from severe retrograde amnesia, while learning of new information and delayed recall after 4 h was normal. After few days, however, the patient was no longer able to recall any aspect of the previously accessible memories. This reflects what has been recently termed accelerated long-term forgetting (25). In contrast to the case report by De Renzi and Lucchelli, most studies demonstrated accelerated long-term forgetting in patients with epilepsy, most of them suffering from complex partial seizures originating from the temporal lobe (26). In one case, accelerated long-term forgetting was observed in a patient with (paraneoplastic) limbic encephalitis with complex partial seizures (27). Parallel to the currently presented case report, this patient also reported a preceding behavioral change including depression and sleep abnormalities. Accelerated long-term forgetting has also been demonstrated in pediatric patients with idiopathic epilepsy (28, 29). The mechanism underlying accelerated long-term forgetting still remains obscure, but ictal and interictal epileptic activity as well as mesiotemporal pathologies have been discussed as etiological factors (25).

In the case report at hand, the reported subjective relief from accelerated long-term forgetting led to vast improvement in self-perceived memory, despite some deterioration in objective learning and memory functions (using standard retention intervals of 30 min) at the final assessment (objective assessment of accelerated long-term forgetting was not performed again). Moreover, the reported subjective relief from anterograde accelerated long-term forgetting was accompanied by partial recovery of retrograde autobiographical memory. This points to a partly reversible access problem to recent episodic memories rather than permanent memory loss. In this regard, a role of the active inflammatory process can be assumed beyond the hypothesized effects of epileptic activity.

## Conclusion

Amygdala swelling, retrograde autobiographic memory loss, accelerated long-term forgetting, and emotional instability may serve as indicators of limbic encephalitis even in the absence of overt epileptic seizures. The monitoring of such patients calls for a standardized and concerted multilevel diagnostic approach with repeated assessments. Cognitive and behavioral problems in limbic encephalitis seem to originate from the complex interplay of active inflammation, structural changes, and epileptic dysfunction/seizures, and they appear (in part) reversible when the disease is successfully treated.

## Author Contributions

All authors conceptualized the study. JAW drafted the manuscript. JAW, VLV, GW, and KJL provided figures. All authors revised and extended the manuscript, and approved its final version. All authors agree to be accountable for all aspects of the work.

## Conflict of Interest Statement

Juri-Alexander Witt, Viola Lara Vogt, Guido Widman, Karl-Josef Langen, Christian Erich Elger, and Christoph Helmstaedter report no conflicts of interest.
